# Integrated Analysis Reveals a lncRNA–miRNA–mRNA Network Associated with Pigeon Skeletal Muscle Development

**DOI:** 10.3390/genes12111787

**Published:** 2021-11-11

**Authors:** Tao Zhang, Can Chen, Shushu Han, Lan Chen, Hao Ding, Yueyue Lin, Genxi Zhang, Kaizhou Xie, Jinyu Wang, Guojun Dai

**Affiliations:** 1 College of Animal Science and Technology, Yangzhou University, Yangzhou 225009, China; zhangt@yzu.edu.cn (T.Z.); can19981224@163.com (C.C.); 15138343214@163.com (H.D.); lyy3078539326@163.com (Y.L.); jywang@yzu.edu.cn (J.W.); daigj@yzu.edu.cn (G.D.); 2 Joint International Research Laboratory of Agriculture and Agri-Product Safety of Ministry of Education of China, College of Animal Science and Technology, Yangzhou University, Yangzhou 225009, China; chenlan9326@163.com; 3 Shandong Chengwu County Animal Husbandry Service Center, Department of Agriculture and Rural Affairs of Heze City, Heze 274200, China; cwxmfwzx@gmail.com; 4 College of Veterinary Medicine, Yangzhou University, Yangzhou 225009, China

**Keywords:** pigeon, skeletal muscle development, lncRNA, ceRNA network, miRNA

## Abstract

Growing evidence has demonstrated the emerging role of long non-coding RNA as competitive endogenous RNA (ceRNA) in regulating skeletal muscle development. However, the mechanism of ceRNA regulated by lncRNA in pigeon skeletal muscle development remains unclear. To reveal the function and regulatory mechanisms of lncRNA, we first analyzed the expression profiles of lncRNA, microRNA (miRNA), and mRNA during the development of pigeon skeletal muscle using high-throughput sequencing. We then constructed a lncRNA–miRNA–mRNA ceRNA network based on differentially expressed (DE) lncRNAs, miRNAs, and mRNAs according to the ceRNA hypothesis. Functional enrichment and short time-series expression miner (STEM) analysis were performed to explore the function of the ceRNA network. Hub lncRNA–miRNA–mRNA interactions were identified by connectivity degree and validated using dual-luciferase activity assay. The results showed that a total of 1625 DE lncRNAs, 11,311 DE mRNAs, and 573 DE miRNAs were identified. A ceRNA network containing 9120 lncRNA–miRNA–mRNA interactions was constructed. STEM analysis indicated that the function of the lncRNA-associated ceRNA network might be developmental specific. Functional enrichment analysis identified potential pathways regulating pigeon skeletal muscle development, such as cell cycle and MAPK signaling. Based on the connectivity degree, lncRNAs *TCONS_00066712*, *TCONS_00026594*, *TCONS_00001557*, *TCONS_00001553*, and *TCONS_00003307* were identified as hub genes in the ceRNA network. lncRNA *TCONS_00026594* might regulate the FSHD region gene 1 (*FRG1*)/ SRC proto-oncogene, non-receptor tyrosine kinase (*SRC*) by sponge adsorption of cli-miR-1a-3p to affect the development of pigeon skeletal muscle. Our findings provide a data basis for in-depth elucidation of the lncRNA-associated ceRNA mechanism underlying pigeon skeletal muscle development.

## 1. Introduction

Over the past decades, breeders and growers have devoted themselves to increasing the growth rate of birds, feed efficiency, and size of breast muscle to meet the global increasing demand for poultry meat [1]. Poultry meat has become the most consumed and affordable type of meat among animal sources, worldwide [2]. By 2026, poultry meat is expected to account for 45% of global meat consumption [3]. In recent years, with improvements in living standards, people have begun to pay more attention to meat quality. Pigeon (*Columba livia*) is the fourth-largest poultry product in China. Nowadays, pigeon meat is consumed as high-quality and nutritional poultry meat and is gaining popularity among consumers, globally [4]. Pigeon meat is characterized by a high nutritive value with low cholesterol and relatively high protein content compared with other poultry, and it can be used as a valuable, inclusive component of the human diet [5]. Modern consumers are increasingly aware of the relationships between diet and health, resulting in an increasing demand for pigeon meat. Accordingly, the pigeon industry is now focusing on the increasing growth rate of pigeons to enhance the industry’s capacity to increase production and profitability [6].

Meat production, one of the important indicators to measure the economic value of meat pigeons, is determined by the growth and development of skeletal muscle [7]. Therefore, elucidating the molecular mechanisms that regulate the growth and development of skeletal muscle is an essential prerequisite for improving the meat production of pigeons by molecular breeding technology. However, molecular mechanisms regulating pigeon skeletal muscle myogenesis remain largely unknown compared with other poultry. Non-coding RNA (ncRNA) is a class of RNA that generally does not encode a protein, including long non-coding RNA (lncRNA), microRNA (miRNA), circular RNA, and piwi-interacting RNA, many of which are known to function as regulators of transcription [8]. Skeletal muscle growth and development are highly sophisticated and complex biological processes regulated by multiple factors and signal pathways [9,10]. Recent studies have confirmed the essential roles of lncRNA and miRNA in regulating poultry skeletal muscle myogenesis [11,12,13,14,15,16]. Nevertheless, there is no research on the roles of lncRNA and miRNA in regulating pigeon skeletal muscle development. 

In 2011, Salmena et al. proposed the competitive endogenous RNA (ceRNA) hypothesis that protein-coding RNAs and lncRNAs can act as ceRNAs to communicate by competitively binding to miRNAs sites [17,18]. According to the ceRNA hypothesis, many investigators have devoted themselves to elucidating the ceRNA roles of lncRNAs in skeletal muscle myogenesis by constructing ceRNA networks [19,20,21,22]. Studies have shown that lncRNA can regulate skeletal muscle myogenesis of poultry and livestock by acting as ceRNA [23,24,25]. However, the regulatory mechanisms of lncRNA as ceRNA, and the lncRNA-associated ceRNA network involved in skeletal muscle development of pigeons remain elusive.

In the present study, we first characterized the expression profiles of lncRNA, miRNA, and mRNA during the development of pigeon skeletal muscle by high-throughput RNA sequencing (RNA-seq). The differentially expressed (DE) lncRNAs, miRNAs, and mRNAs were then identified. Based on the ceRNA hypothesis, a lncRNA–miRNA–mRNA ceRNA network was constructed by correlation analysis and target prediction. Our study aims to construct a lncRNA-associated ceRNA network and thereby identify key lncRNA–miRNA–mRNA interactions involved in pigeon skeletal muscle development, which will enhance our understanding of the molecular mechanisms underlying pigeon skeletal muscle myogenesis.

## 2. Materials and Methods

### 2.1. Sample Preparation

The White King pigeons used in this study were obtained from Wuxi Sanxiangan Agricultural Technology Development Co., Ltd. (Wuxi, China). The pigeon breast muscle tissues of the 8-day-old embryo (E8), the 13-day-old embryo (E13), 1-day-old (D1), and 10-day-old (D10) were collected. Each period contained three biological replicates. All tissue samples were flash-frozen in liquid nitrogen and stored at −80 °C. Total RNA was isolated using Trizol reagent (Invitrogen, CA, USA) following the manufacturer’s protocol. RNA quality was assessed on an Agilent 2100 Bioanalyzer (Agilent Technologies, city, Santa Clara, CA, USA) and checked using RNase-free agarose gel electrophoresis.

### 2.2. LncRNA Library Construction and Sequencing

After the total RNA was extracted, ribosome RNAs (rRNAs) were removed, and mRNAs and ncRNAs were enriched. The enriched mRNAs and ncRNAs were fragmented into short fragments using a fragmentation buffer and reverse transcribed into cDNA with random primers. DNA polymerase I, RNase H, dNTP (dUTP instead of dTTP), and buffer were used to synthesize second-strand cDNA. The cDNA fragments were then purified with a QiaQuick PCR extraction kit (QIAGEN, Hilden, Germany). The purified products were end-repaired, poly(A)-added, and ligated to Illumina sequencing adapters. Then, UNG (Uracil-N-Glycosylase) was used to digest the second-strand cDNA. The digested products were size selected by agarose gel electrophoresis, PCR amplified, and sequenced using Illumina HiSeq^TM^ 4000 by Gene Denovo Biotechnology Co. (Guangzhou, China). 

### 2.3. miRNA Library Construction and Sequencing

After the total RNA was isolated, small RNAs in a size range of 18–30 nt were enriched by polyacrylamide gel electrophoresis (PAGE). Then, the 3’ adapters were added, and the 36–44 nt long RNAs were enriched. The 5’ adapters were then ligated to the RNAs as well. The ligation products were reverse transcribed by PCR amplification, and the 140–160 bp size PCR products were enriched to generate a cDNA library and sequenced using Illumina HiSeq^TM^ 2500 by Gene Denovo Biotechnology Co. (Guangzhou, China).

### 2.4. LncRNA and mRNA Identification

High-quality clean reads were obtained by removing reads containing adapters, removing reads containing more than 10% of unknown nucleotides (N), and removing low quality reads containing more than 50% of low quality (*Q*-value ≤ 20) bases using fastp (version 0.18.0) with parameters set as a default [26]. Then, the high-quality clean reads were mapped to the rRNA database to remove rRNA mapped reads using Bowtie2 with 0 mismatches [27]. The remaining reads were mapped to the Columba livia reference genome (assembly Cliv_1.0) using HISAT2 (version 2.1.0) with “-RNA-strandness RF” and other parameters set as default [28]. The mapping percentage ranged from 83.35% to 85.32%, with an average of 84.45%. Transcripts in each sample were assembled and quantified using StringTie (version 1.3.4) with default parameters [29]. The lncRNAs were predicted based on the novel transcripts using CNCI [7] (version 2) and CPC [8] (version 0.9-r2) (http://cpc.cbi.pku.edu.cn/, accessed on 15 September 2020) software by default parameters. FPKM (fragment per kilobase of transcript per million mapped reads) value was calculated to quantify the expression abundance of lncRNA and mRNA using StringTie (version 1.3.4) with default parameters [29].

### 2.5. miRNA Identification

Quality control was performed using FastQC with default parameters (http://www.bioinformatics.bbsrc.ac.uk/projects/fastqc, accessed on 16 September 2020). Clean tags were obtained by removing low-quality reads containing more than one low quality (*Q*-value ≤ 20) base or containing unknown nucleotides (N), removing reads without 3’ adapters, removing reads containing 5’ adapters, removing reads containing 3’ and 5’ adapters but without small RNA fragment between them, removing reads containing ployA in small RNA fragment, and removing reads shorter than 18 nt (not include adapters). The clean tags were aligned with small RNAs in the GenBank database (Release 209.0) and Rfam database (Release 11.0) to identify and remove rRNA, scRNA, snoRNA, snRNA and tRNA using blastall (version 2.2.25) (https://www.ncbi.nlm.nih.gov/Class/BLAST/, accessed on 16 September 2020). Meanwhile, the clean tags were mapped to the Columba livia reference genome (assembly Cliv_1.0) to remove fragments from mRNA degradation using bowtie2 with “-v 0 --best --strata -a” and other parameters set as a default [27]. The mapping percentage ranged from 63.51% to 79.93%, with an average of 75.64%. The existing miRNAs and known miRNAs were then identified by aligning filtered clean tags against the miRbase database (Release 22) using Bowtie2 with “-v 0 --best --strata -a”. The novel miRNAs were predicted by Mireap_v0.2 based on the unannotated tags with parameters set as a default (http://sourceforge.net/projects/mireap/, accessed on 16 September 2020). The miRNA expression level was calculated and normalized to transcripts per million (TPM).

### 2.6. Differentially Expression Analysis

DE lncRNAs and DE mRNAs were identified using the negative binomial generalized linear model implemented in DESeq2 software with thresholds false discovery rate (FDR) < 0.05 and |log2 fold change (FC)| > 1 [30]. DE miRNAs were identified using the generalized linear model implemented in edgeR [31] software with thresholds FDR < 0.05 and |log2FC| > 1. The DE lncRNAs, DE mRNAs, and DE miRNAs were obtained by the six pairwise comparisons: E8 vs. E13, E8 vs. D1, E8 vs. D10, E13 vs. D1, E13 vs. D10, and D1 vs. D10.

### 2.7. ceRNA Network Construction

**Figure 1:**
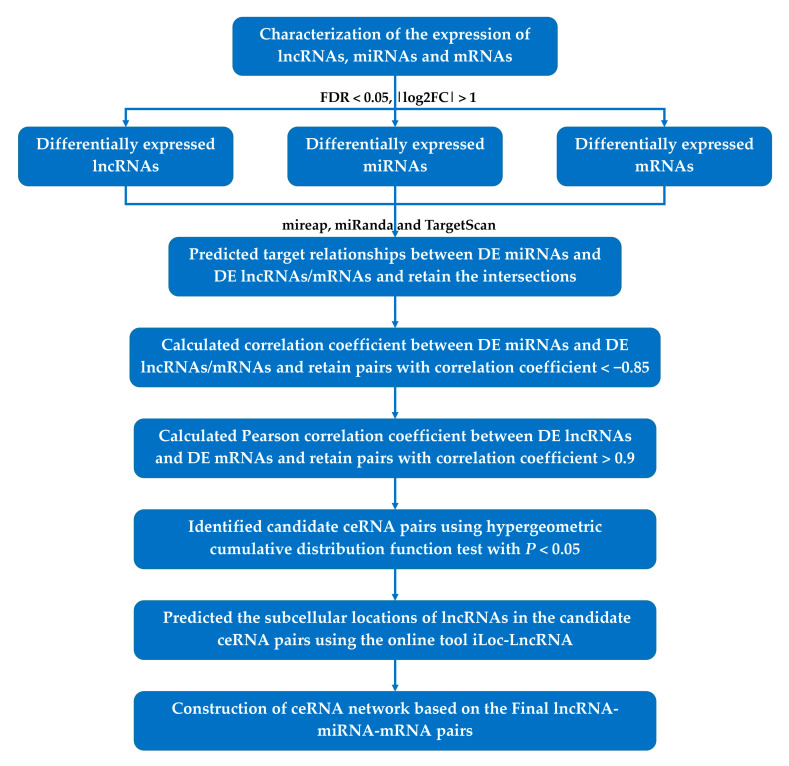
ceRNA network construction flowchart. FDR: false discovery rate, log2FC: log2 fold change, DE: differentially expressed, ceRNA: competitive endogenous RNA, lncRNA: long non-coding RNA, miRNA: microRNA, mRNA: messenger RNA.

The lncRNA-associated ceRNA network was constructed based on all DE lncRNAs, DE miRNAs, and DE mRNAs according to the ceRNA hypothesis (Figure 1) [17,32]. We firstly predicted the target relationships between DE miRNAs and DE lncRNAs/mRNAs using mireap (http://sourceforge.net/projects/mireap/, accessed on 4 February 2021), miRanda [33], and TargetScan [34] software, with parameters listed in Table S1. The intersection of the predicted results obtained by three software was considered the final predicted results of target relationships. Secondly, we calculated Spearman’s rank correlation coefficient between DE miRNA and DE lncRNA/mRNA expression profiles. miRNA–lncRNA and miRNA–mRNA pairs with the correlation coefficient<−0.85 were selected for subsequent analysis. Thirdly, we calculated the Pearson correlation coefficients (PCC) between the expression profiles of DE lncRNA and DE mRNA targeted by common miRNA. Pairs with PCC > 0.9 were selected as potential co-expressed ceRNA pairs. Fourthly, we used the hypergeometric cumulative distribution function test to examine whether the common miRNA sponges between the two genes were significant. Pairs with a *p* < 0.05 were selected as candidate ceRNA pairs. Similar to proteins, the function of lncRNAs depends on their subcellular localization. According to the ceRNA hypothesis, whether lncRNAs act as effective ceRNAs depends mainly on their abundance and subcellular localization in the cytoplasm [35,36]. Therefore, we predicted the subcellular locations of lncRNAs in the candidate ceRNA pairs using the online tool iLoc-LncRNA [37], and pairs including lncRNAs located in the cytoplasm were considered final ceRNA pairs. The constructed lncRNA-associated ceRNA network was visualized using Gephi (Version 0.9.2) software (https://gephi.org/, accessed on 12 August 2020). 

### 2.8. Functional Enrichment Analysis of the ceRNA Network

Short time-series expression miner (STEM) analysis of mRNAs involved in the ceRNA network was performed using Omicsmart, a real-time interactive online platform for data analysis (http://www.omicsmart.com, accessed on 30 October 2021). Gene ontology (GO) annotation and Kyoto encyclopedia of genes and genome (KEGG) enrichment analysis was performed using the R package clusterProfiler 4.0 [38]. A *p* < 0.05 was considered significant.

### 2.9. Identification of Hub lncRNA–miRNA–mRNA Interactions

Genes with high connectivity degrees are termed “hub genes” and are usually functionally important in a network [39]. In the present study, the top five lncRNAs ranked by connectivity degree were considered hub lncRNAs. LncRNA–miRNA–mRNA interactions containing hub lncRNA were selected as potential ceRNA interactions. The subnetwork includes the hub ceRNA interactions was visualized using Gephi (Version 0.9.2) software (https://gephi.org/, accessed on 12 August 2020).

### 2.10. Validation of the Expression of ceRNA Interactions Using qRT-PCR

The specific qRT-PCR primers of lncRNAs were designed using the online NCBI Primer-BLAST tool (https://www.ncbi.nlm.nih.gov/tools/primer-blast/, accessed on 12 April 2021). The miRNA stem-loop primer and miRNA RT-qPCR primers were designed using miRNA Design software (version 1.01) (Vazyme, Nanjing, China). The primer sequences were listed in Table S2. miRNA was quantified using the Universal SYBR qPCR Master Mix Kit (Vazyme, Nanjing, China), while mRNA was quantified using the HiScript® II One Step qRT-PCR SYBR Green Kit (Vazyme, Nanjing, China). Gene expression was calculated according to the 2-ΔΔCT (2^−ΔΔCt^) method [40].

### 2.11. Dual-Luciferase Activity Assay

When the cell confluence reached about 60%, the NC mimics, miR-1a-3p mimics, pmirGLO+, pmirGLO+TCONS_00026594-WT/-Mu, pmirGLO+FRG1 3’ UTR-WT/-Mu, pmirGLO+SRC 3’ UTR-WT/-Mu, and pmirGLO+FMNL2 3’ UTR-WT/-Mu were co-transfected into DF1 cells (Table S3). After incubation for 24 h, the cells were collected and lysed, and dual-luciferase activity was measured using a dual-luciferase assay kit (Vazyme, Nanjing, China) and using a PerkinElmer EnSpire Multilabel Reader 2300 (PerkinElmer, Waltham, MA, USA). The firefly luciferase activity was normalized against Renilla luciferase activity.

## 3. Results

### 3.1. Identification of DE lncRNAs, miRNAs, and mRNAs

**Figure 2:**
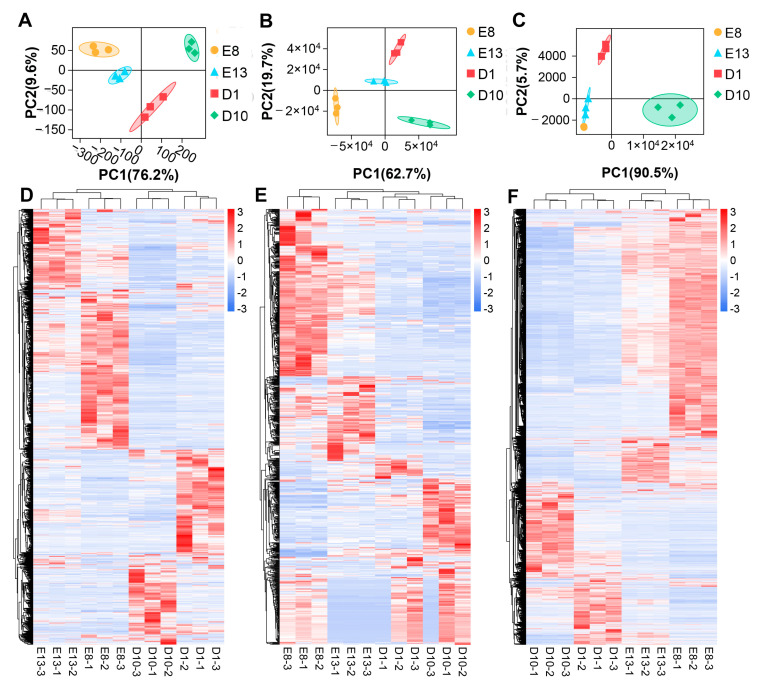
Identification of DE lncRNAs, miRNAs, and mRNAs during pigeon skeletal muscle development: (**A**) PCA plot of all lncRNAs expressed in pigeon skeletal muscle samples; (**B**) PCA plot of all miRNAs expressed in pigeon skeletal muscle samples; (**C**) PCA plot of all mRNAs expressed in pigeon skeletal muscle samples; (**D**) hierarchical clustering heatmap of all the DE lncRNAs; (**E**) hierarchical clustering heatmap of all the DE miRNAs; (**F**) hierarchical clustering heatmap of all the DE mRNAs. Both PCA and hierarchical clustering results demonstrate excellent repeatability of the samples.

By RNA-seq and miRNA-seq, a total of 5076 lncRNAs, 2362 miRNAs, and 32,527 mRNAs were detected in pigeon skeletal muscle samples (Table S4). Figure 2A–C displays the PCA plot of all the samples based on all the detected lncRNAs, miRNA, and mRNA, respectively. The PCA plots show that the samples are mainly clustered based on collection time points, indicating the excellent repeatability of the samples. Based on the expression level comparison, a total of 1625 DE lncRNAs, 11,311 DE mRNAs, and 573 DE miRNAs were identified between samples of different developmental ages with a threshold of FDR < 0.05 and |log_2_FC| > 1 (Table S5, Figure S1). Likewise, hierarchical clustering heatmap analysis of DE lncRNA, miRNAs, and mRNAs revealed distinct gene expression profiles between different groups (Figure 2D–F).

### 3.2. Construction of lncRNA-Associated ceRNA Network

By target relationship prediction, a total of 89,093 miRNA–mRNA pairs were obtained, including 573 DE miRNAs and 4051 DE mRNAs. Meanwhile, 133,751 miRNA–lncRNA pairs were generated, consisting of 573 DE miRNAs and 1625 DE lncRNAs. Then, miRNA–mRNA and miRNA–lncRNA pairs with Spearman’s rank correlation coefficient< −0.85 were selected as candidate miRNA–ceRNA pairs. A total of 2681 miRNA–mRNA pairs and 1838 miRNA–lncRNA pairs were identified (Table S6). According to the ceRNA hypothesis, there should be a significant positive correlated expression between ceRNAs (lncRNAs and mRNAs) competing for the same miRNA response elements (MREs). Thus, we calculated the PCC between the expression profiles of mRNAs involved in the 2681 miRNA–mRNA pairs and lncRNAs involved in the 1838 miRNA–lncRNA pairs. With a PCC > 0.9, 51,691 potential ceRNA pairs were identified, containing 507 lncRNAs and 1089 mRNAs (Table S7). A hypergeometric cumulative distribution function test was performed to verify the 51,691 potential ceRNA pairs further. A total of 9599 ceRNAs (lncRNA–mRNA) were identified with a *p* < 0.05, comprising 386 lncRNAs and 850 mRNAs (Table S8). Subcellular location prediction showed that 259 lncRNAs were predicted to be located in the cytoplasm, interacting with 153 miRNAs and 775 mRNAs, forming 9120 lncRNA–miRNA–mRNA ceRNA interactions (Table S9). The ceRNA network was constructed based on the 9120 lncRNA–miRNA–mRNA ceRNA interactions using Gephi software. Figure S2 illustrates the constructed lncRNA–miRNA–mRNA ceRNA network related to pigeon skeletal muscle development.

### 3.3. Functional Analysis of the ceRNA Network

**Figure 3:**
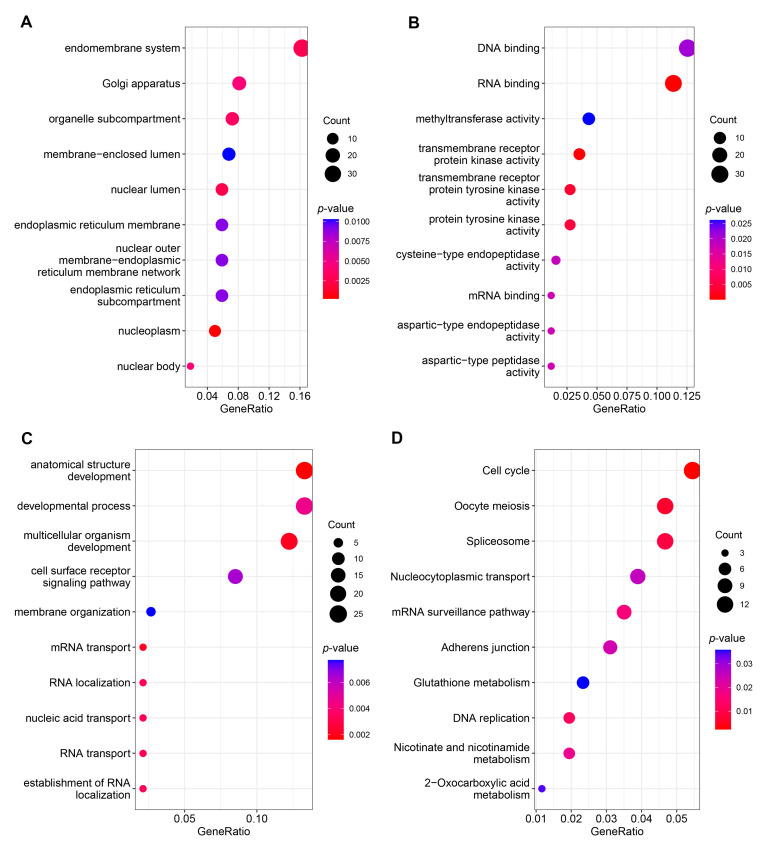
GO and KEGG enrichment analysis of mRNAs involved in the ceRNA network: (**A**) ScatTable 10 significant cellular component terms; (**B**) scatter plot of the top 10 significant molecular function terms; (**C**) scatter plot of the top 10 significant biological process terms; (**D**) scatter plot of the top 10 significant pathways. The node size is proportional to the gene number. The color from red to blue indicates the *p*-value from low to high.

DE mRNAs in the constructed ceRNA network were subjected to GO and KEGG pathway enrichment analysis to better understand the function of the network. GO enrichment analysis showed that 74 terms were significantly enriched (*p* < 0.05), including 27 cellular component terms, 18 molecular function terms, and 29 biological process terms (Figure 3A–C) (Table S10). The top three significant terms were anatomical structure development, multicellular organism development, and mRNA transport in the biological process category. In the molecular function category, the top three terms were RNA binding, transmembrane receptor protein kinase activity, and transmembrane receptor protein tyrosine kinase activity. Moreover, nucleoplasm, nuclear lumen, and endomembrane system were the top three significant terms in the cellular component category. KEGG pathway analysis identified 12 significantly enriched pathways such as cell cycle, oocyte meiosis, and spliceosome (Figure 3D) (Table S11).

### 3.4. Short Time-Series Expression Miner and Function Analysis

**Figure 4:**
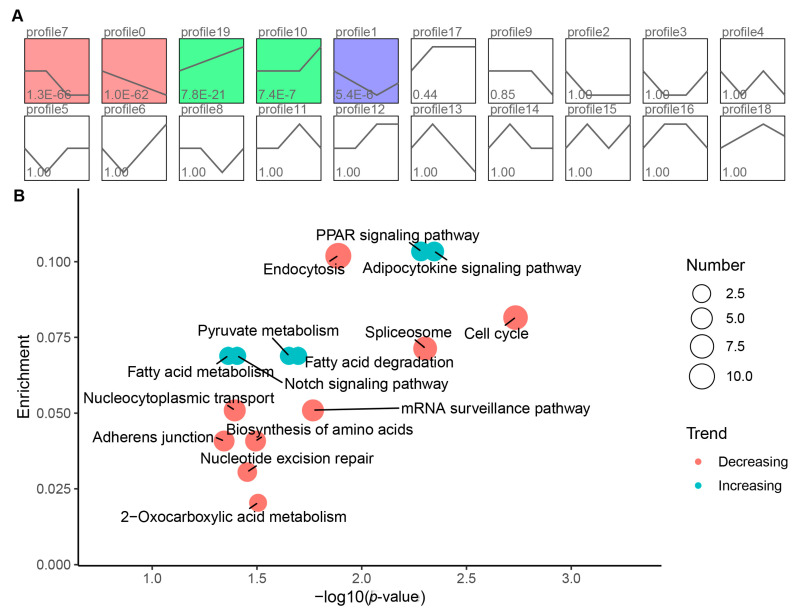
Trend and function enrichment analysis of mRNAs in the ceRNA network: (**A**) trend analysis of mRNAs in the ceRNA network using STEM. The significantly enriched profiles are purple, green, and red colored (*p* < 0.05). Colors represent the categories clustered by gene profiles; (**B**) scatter plot of significantly enriched KEGG pathways of mRNAs in the increasing and decreasing profiles. The node size is proportional to the gene number. Red and turquoise represent pathways of decreasing and increasing profiles, respectively.

To better understand the ceRNA network, we performed a STEM analysis on the 775 mRNAs involved in the ceRNA networks. The results demonstrated that five significant enriched profiles were identified (*p* < 0.05) and could be classified into two categories: increasing (profile 10 and 19) and decreasing (profile 0, 1, and 7) (Figure 4A). The expression of genes in the increasing profile was upregulated, while genes in the decreasing profiles were downregulated during skeletal muscle development (Table S12). The top three significant genes in the increasing profiles were A306_00002783, matrix Gla protein (*MGP*), and ryanodine receptor 3 (*RYR3*). While they were fibroblast growth factor receptor 2 (*FGFR2*), HECT domain E3 ubiquitin-protein ligase 1 (*HECTD1*), and retinoic acid receptor beta (*RARB*) in the decreasing profiles. We then performed KEGG analysis on genes in the increasing and decreasing profiles, respectively. KEGG analysis of genes in the increasing profile identified six significantly enriched pathways, including adipocytokine signaling pathway, peroxisome proliferators-activated receptor (PPAR) signaling pathway, and fatty acid degradation pathway. At the same time, nine significantly enriched pathways, including cell cycle, spliceosome, and endocytosis, were identified for genes in the decreasing profiles (Figure 4B).

### 3.5. Identification of Crucial lncRNA–miRNA–mRNA Interactions

**Figure 5:**
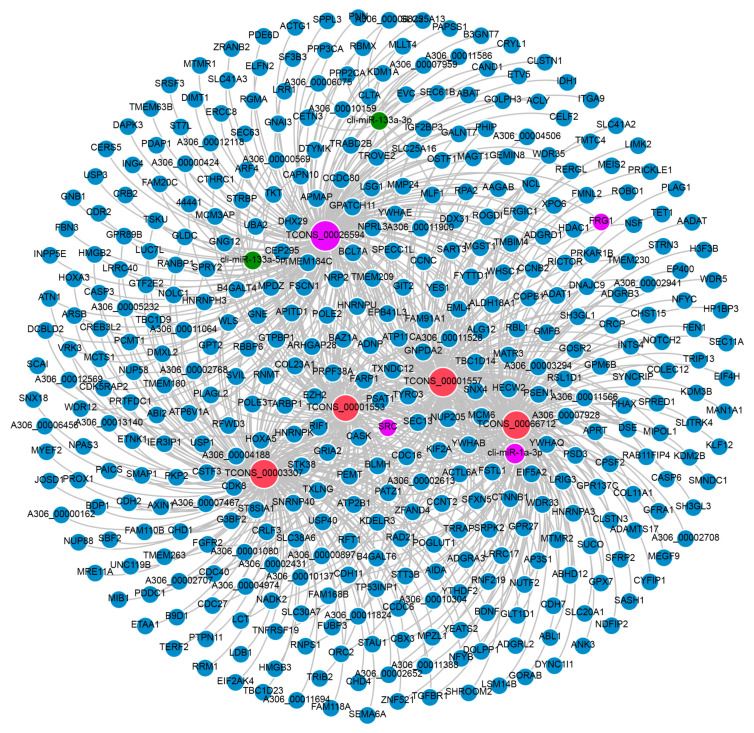
Visualization of the 136 crucial lncRNA–miRNA–mRNA interactions. Red, green, and blue nodes represent lncRNA, miRNA, and mRNA, respectively. Pink nodes represent the ceRNA interactions validated by dual-luciferase assay. The node size is proportional to the connectivity degree.

**Figure 6:**
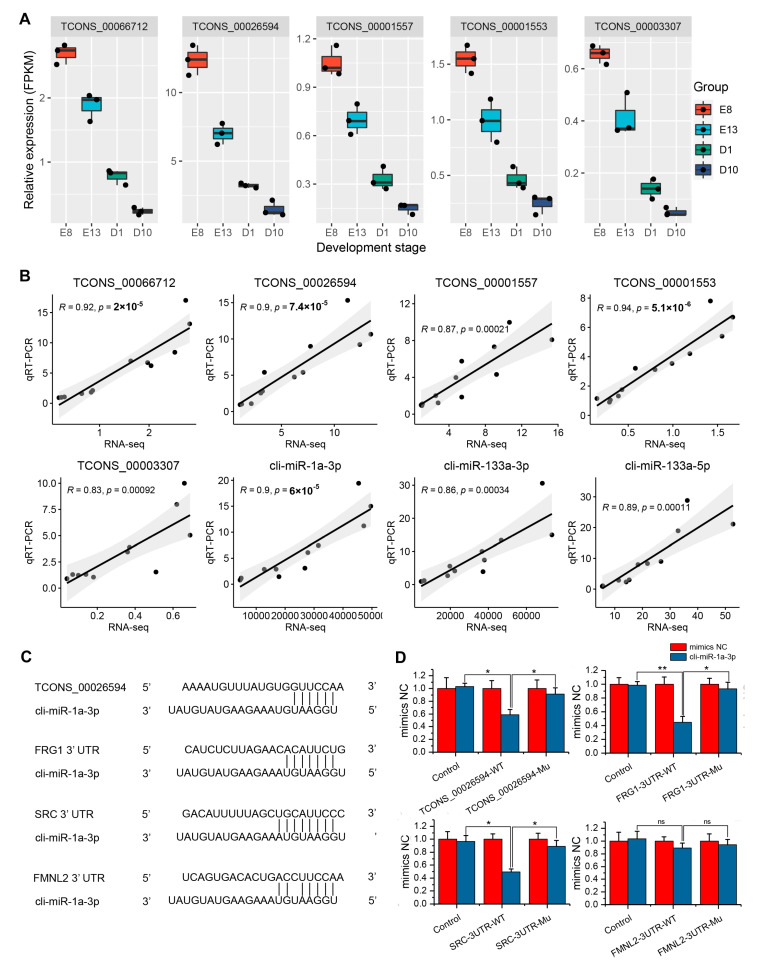
Identification of potential lncRNA–miRNA–mRNA interactions in pigeon skeletal muscle development: (**A**) expression profiles of the five hub lncRNAs during skeletal muscle development; (**B**) correlation analysis of expression profiles of the five hub lncRNAs and three muscle-specific miRNAs detected by RNA-seq and qRT-PCR. *p* < 0.05 indicates significant difference; (**C**) target relationship prediction between cli-miR-1a-3p and *TCONS_00026594* as well as *FRG1*, *SRC*, and *FMNL2*; (D) validation of the target relationships between cli-miR-1a-3p and *TCONS_00026594*/*FRG1*/*SRC* using dual-luciferase assay. * *p* < 0.05, ** *p* < 0.01, ns *p* > 0.05.

To identify crucial lncRNA–miRNA–mRNA interactions associated with pigeon skeletal muscle development, we calculated the connectivity of each node in the ceRNA network using Gephi software. The top five lncRNAs with the highest connectivity degree were classified as hub lncRNAs, including *TCONS_00066712*, *TCONS_00026594*, *TCONS_00001557*, *TCONS_00001553*, and *TCONS_00003307* (Figure 5). All the five hub lncRNAs were highly expressed during the embryonic stage and then were downregulated with skeletal muscle development (Figure 6A). The five hub lncRNAs interact with 29 miRNAs and 404 mRNAs, forming 1332 lncRNA–miRNA–mRNA ceRNA interactions (Table S13). We then performed KEGG enrichment analysis on the 404 mRNAs targeted by the 29 miRNAs in the ceRNA network (Table S14). In total, 11 KEGG pathways were significantly enriched, containing cell cycle, spliceosome, and nucleocytoplasmic transport. Cell cycle was the top enriched pathway consisting of 12 genes of ABL proto-oncogene 1, non-receptor tyrosine kinase (*ABL1*), cyclin B2 (*CCNB2*), cell division cycle 16 (*CDC16*), cell division cycle 27 (*CDC27*), histone deacetylase 1 (*HDAC1*), mini-chromosome maintenance complex component 6 (*MCM6*), origin recognition complex subunit 2 (*ORC2*), RAD21 cohesin complex component (*RAD21*), RB transcriptional corepressor like 1 (*RBL1*), tyrosine 3-monooxygenase/tryptophan 5-monooxygenase activation protein beta (*YWHAB*), tyrosine 3-monooxygenase/tryptophan 5-monooxygenase activation protein epsilon (*YWHAE*), and tyrosine 3-monooxygenase/tryptophan 5-monooxygenase activation protein theta (*YWHAQ*). It was also found that many muscle-specific miRNAs interacted with the five hub lncRNAs, such as cli-miR-133a-3p, cli-miR-133a-5p, and cli-miR-1a-3p, generating 136 lncRNA–miRNA–mRNA interactions (Figure 5). These 136 interactions were identified as potential crucial ceRNA pairs regulating pigeon skeletal muscle development.

### 3.6. Validation of ceRNA Interactions Using qRT-PCR and Dual-Luciferase Assay

We performed qRT-PCR to validate the expression of the five hub lncRNAs and the three muscle-specific miRNAs cli-miR-133a-3p, cli-miR-133a-5p, and cli-miR-1a-3p. As shown in Figure 6B, five hub lncRNAs and three muscle-specific miRNAs exhibited concordant expression profiles in RNA-seq and qRT-PCR analysis. Studies have shown that miR-1, FSHD region gene 1 (*FRG1*), SRC proto-oncogene, non-receptor tyrosine kinase (*SRC*), and formin-like 2 (*FMNL2*) regulate myogenesis [41,42,43,44]. We found that lncRNA *TCONS_00026594*, cli-miR-1a-3p, *FRG1*, *SRC*, and *FMNL2* showed potential ceRNA interactions. qRT-PCR results also showed concordant expression profiles of *FRG1*, *SRC,* and *FMNL2* with RNA-seq results (Figure S3). Therefore, we validated the interaction mechanisms of the *TCONS_00026594*–cli-miR-1a-3p–*FRG1*/*SRC*/*FMNL2* axis identified from the lncRNA-associated ceRNA network by target prediction and dual-luciferase assay. As shown in Figure 6C, cli-miR-1a-3p could target lncRNA *TCONS_00026594*, as well as *FRG1*, *SRC*, and *FMNL2* 3’ UTR regions. The dual-luciferase reporter showed significant decreases in luciferase activity of the *TCONS_00026594*, *FRG1*, and *SRC* wild type, and luciferase activity was restored by the *TCONS_00026594*, *FRG1*, and *SRC* mutant sequence, which determined the target relationship between cli-miR-1a-3p and *TCONS_00026594*/*FRG1*/*SRC*. No significant difference in luciferase activity was observed between the *FMNL2* wild-type and mutant sequence (Figure 6D). 

## 4. Discussion

Poultry skeletal muscle development is a complex and tightly regulated developmental process comprising myoblast differentiation from the mesodermal precursor cells to form mature myotubes. Genetic factors such as transcription factors, gene polymorphism, DNA methylation, and non-coding RNAs (ncRNAs) work together to control skeletal muscle development [45,46]. Recent studies have confirmed the essential roles of lncRNA in regulating skeletal muscle development [23,24,25]. However, the expression of lncRNAs in pigeon skeletal muscle remains unknown. To our knowledge, no studies exploring the potential role of lncRNA in pigeon muscle development have been reported to date. This study identified 5076 lncRNAs in 12 pigeon skeletal muscle samples of different developmental stages, laying a foundation for further functional studies of lncRNA in skeletal muscle myogenesis.

The ceRNA hypothesis has attracted much attention in recent years. Accumulating evidence suggests that lncRNA may act as a ceRNA for particular miRNAs to modulate the target genes of the miRNAs [47]. The ceRNA hypothesis provides a new perspective in terms of studying the role and regulatory mechanism of lncRNA in skeletal muscle development. An increasing number of research aiming to construct lncRNA-associated ceRNA networks in myogenesis has been conducted [20,48,49]. The involvement of lncRNA as ceRNA in regulating animal skeletal muscle development has also been described [23,24,25,50,51]. Therefore, constructing a ceRNA network may help elucidate the regulatory mechanisms underlying the development of pigeon skeletal muscle. In this study, we first identified 1625 DE lncRNAs, 573 DE miRNAs, and 11,311 DE mRNAs between muscle samples of different developmental ages. Based on target prediction, correlation analysis, and subcellular location prediction, we finally constructed a lncRNA–miRNA–mRNA ceRNA network consisting of 259 lncRNAs, 153 miRNAs, and 775 mRNAs, forming 9120 lncRNA–miRNA–mRNA ceRNA interactions. It should be noted that, according to the ceRNA hypothesis, whether lncRNAs act as effective ceRNAs depends mainly on their abundance and subcellular localization in the cytoplasm [35,36]. However, most studies focusing on constructing ceRNA networks have ignored the effect of localization on the way lncRNA exerts its function. Thus, lncRNA, which is mainly localized in the nucleus, may be included, which will hinder the reliability of the ceRNA network. Our study predicted the subcellular localization of the lncRNAs, and only lncRNAs that were predicted to be mainly localized in the cytoplasm were retained in the ceRNA network. In comparison with previous studies [20,48,49], our study constructed a lncRNA-associated ceRNA network with higher reliability. 

Avian skeletal muscle growth is comprised of distinct and precisely regulated periods of embryonic and post-hatch muscle development [52]. Studies have shown that lncRNA and miRNA expression is usually tissue specific or affects specific developmental stages [53,54]. Therefore, we questioned whether the function of the constructed ceRNA network possesses development specific to regulating pigeon muscle development. We then performed a STEM analysis to cluster the expression profiles of mRNA in the ceRNA network. The STEM analysis identified five significantly enriched profiles that could be divided into two interesting categories: increasing and decreasing, demonstrating a development-specific expression of the genes in the network. Genes in the decreasing profiles showed high expression in the embryonic stage and were significantly downregulated with skeletal muscle development, suggesting their potential roles in embryonic muscle development. The top three significant genes in the decreasing profiles were *FGFR2*, *HECTD1,* and *RARB*. *FGFR2* is a member of the fibroblast growth factor receptor (FGFR) family. *FGFR2* plays a crucial role in the myogenesis of skeletal muscle stem cells [55]. A recent study showed that miR-217-5p regulates myogenesis in skeletal muscle stem cells by targeting *FGFR2* [56]. *RARB* is a target of muscle-specific miRNA miR-1/206 and might be involved in C2C12 myoblast differentiation [57]. *HECTD1* is indispensable for normal embryogenesis and fetal survival [58]. However, there are no reports showing the involvement in skeletal muscle development. Pathway enrichment analysis of mRNAs in the decreasing profiles identified seven significantly enriched pathways that belong to cellular processes and genetic information processing classes, including endocytosis, adherens junction, cell cycle, spliceosome, nucleocytoplasmic transport, nucleotide excision repair, and mRNA surveillance pathway. Skeletal muscle development in avian embryos depends on the proliferation and differentiation of embryonic myoblasts [59]. The above seven pathways might regulate the proliferation and differentiation of embryonic myoblasts, which, in turn, affects embryonic skeletal muscle development in pigeons. 

Genes in the increasing profiles are lowly expressed at the embryonic stage and significantly upregulated following birth, suggesting their potential roles in post-hatch muscle development. The top three significant genes in the increasing profiles were *A306_00002783*, *MGP*, and *RYR3*. *MGP* is a well-known inhibitor of calcification in soft tissues and has been reported to be highly upregulated during bovine myogenesis [60]. *MGP* is confirmed to regulate the myogenic program through inhibiting myostatin (*MSTN*) functionally by disrupting its binding to the receptor [61]. *RYR3* is a ubiquitous calcium release channel detected in the microsomal fractions of differentiated skeletal muscle cells but not in undifferentiated cells. However, *RYR3* was expressed independent of cell fusion and myotube formation [62], and its role in skeletal muscle development remains unclear. Pathway enrichment analysis showed that genes in the increasing profiles were involved in eleven significantly enriched pathways, including myogenesis-related pathways, such as insulin signaling and MAPK signaling [63,64]. Interestingly, we found that four lipid metabolism-related pathways were significantly enriched, containing adipocytokine signaling pathway, PPAR signaling pathway, fatty acid metabolism, and fatty acid degradation. With the development of the skeletal muscle, the intramuscular fat deposition will be accelerated. The accelerated intramuscular fat deposition might be regulated by the activation of the adipocytokine signaling, PPAR signaling, fatty acid metabolism, and fatty acid degradation pathways. 

Hub genes, defined as genes with high connectivity in a module, are considered to be functionally significant [65]. By the connectivity degree, we identified *TCONS_00066712*, *TCONS_00026594*, *TCONS_00001557*, *TCONS_00001553*, and *TCONS_00003307* as hub lncRNAs. Five hub lncRNAs interact with 29 miRNAs and 404 mRNAs, forming a subnetwork consisting of 1332 lncRNA–miRNA–mRNA ceRNA interactions. KEGG pathway enrichment analysis of the 404 mRNAs in the subnetwork showed that the cell cycle was the top enriched pathway, implying that the hub lncRNAs might regulate pigeon skeletal muscle development through the cell cycle pathway. Furthermore, we found that the hub lncRNAs interact with three muscle-specific miRNAs cli-miR-133a-3p, cli-miR-133a-5p and cli-miR-1a-3p [44]. miR-1 and miR-133, which are clustered on the same chromosomal loci, have been confirmed to regulate animal myogenesis by targeting myogenic differentiation 1 (*MYOD1*), myogenin (*MYOG*), serum response factor (*SRF*), myocyte enhancer factor 2 (*MEF2*), transforming growth factor-β (*TGFB*), mechanistic target of rapamycin kinase (*MTOR*), nuclear factor-κB (*NF-κB*) and YY1 transcription factor (*YY1*) [66]. Therefore, cli-miR-133a-3p, cli-miR-133a-5p, and cli-miR-1a-3p might be important factors that mediate the functions of the hub lncRNAs on pigeon skeletal muscle development. Among the five hub lncRNAs, *TCONS_00026594* showed the highest expression level in skeletal muscle. *TCONS_00026594* interacts with miR-1 and three known myogenesis-related genes *FRG1*, *SRC*, and *FMNL2*. *FRG1* is a candidate gene responsible for facioscapulohumeral muscular dystrophy and it is critical for muscle development [67]. Neguembor et al. found that FRG1 binds Suv4-20h1 histone methyltransferase and impairs myogenesis [41]. *SRC* is reported to mediate mechano-activation of TNFα-converting enzyme (*TACE*) and myogenesis in mice [42]. *FMNL2* also plays important role in T-complex 11 like 2 (*TCP11L2*) mediated bovine skeletal muscle-derived satellite cell migration and differentiation [43]. The above studies confirmed the involvement of *FRG1*, *SRC*, and *FMNL2* in myogenesis, implying the potential key role of *TCONS_00026594*–cli-miR-1a-3p–*FRG1*/*SRC*/*FMNL2* axis in regulating pigeon skeletal muscle development. Dual-luciferase assay confirmed the target relationships of *TCONS_00026594*–cli-miR-1a-3p–*FRG1*/*SRC* axis, which offers novel clues to elucidate the developmental mechanism of pigeon skeletal muscle in depth.

Although we identified potential lncRNA–miRNA–mRNA interactions involved in pigeon skeletal muscle development by constructing a lncRNA-associated ceRNA network, a limitation in our study should be noted. The potential lncRNA–miRNA–mRNA interactions were identified by RNA-seq and bioinformatics analysis. The target relationships and functions of the lncRNA–miRNA–mRNA interactions in pigeon skeletal muscle development lack experimental validation at the molecular and cellular levels. In the future, further experimental studies should be conducted to validate the target relationships and to explore the functions of the potential lncRNA–miRNA–mRNA interactions in pigeon skeletal muscle development.

## 5. Conclusions

To our knowledge, this is the first study that constructs a lncRNA-associated ceRNA network in pigeon skeletal muscle development. We constructed a ceRNA network containing 9120 lncRNA–miRNA–mRNA interactions. *TCONS_00066712*, *TCONS_00026594*, *TCONS_00001557*, *TCONS_00001553*, and *TCONS_00003307* were identified as hub lncRNAs in the ceRNA network, which might regulate pigeon skeletal muscle development through the cell cycle pathway. Based on targeting relationship validation results, it is reasonable to believe that the *TCONS_00026594*–cli-miR-1a-3p–FRG1/SRC axis is involved in the regulation of pigeon skeletal muscle development.

## Data Availability

The data presented in this study are openly available in Genome Sequence Archive (https://ngdc.cncb.ac.cn/gsa/ accessed on 16 September 2020). Reference Numbers CRA005062 and CRA005074.

## Supplementary Materials

The following are available online at https://www.mdpi.com/article/10.3390/genes12111787/s1, Table S1: The miRNA stem-loop primer and miRNA RT-qPCR primers, Table S2: The experimental grouping of dual-luciferase activity assay, Table S3: The miRNA–mRNA and miRNA–lncRNA pairs, Table S4: The 9,656 ceRNAs (lncRNA–mRNA) identified by a hypergeometric cumulative distribution function test, Table S5: The final ceRNA (lncRNA–miRNA–mRNA) interactions, Table S6: GO enrichment analysis of the DE mRNAs involved in the ceRNA network, Table S7: KEGG pathway enrichment analysis of the DE mRNAs involved in the ceRNA network, Table S8: The short time-series expression miner analysis on the DE mRNAs involved in the ceRNA networks, Table S9: KEGG pathway enrichment analysis of mRNA interacted with the hub lncRNAs, Table S10: GO enrichment analysis of DE mRNAs involved in the ceRNA network, Table S11: KEGG pathway analysis of DE mRNAs involved in the ceRNA network, Table S12: Short time-series expression miner analysis of DE mRNAs involved in the ceRNA network, Table S13: lncRNA–miRNA–mRNA interactions including the five hub lncRNAs, Table S14: KEGG enrichment analysis of DE mRNAs in the subnetwork, Figure S1: Volcano plot of DE lncRNAs, miRNAs, and mRNAs, Figure S2: The constructed lncRNA–miRNA–mRNA ceRNA network related to pigeon skeletal muscle development, Figure S3: Correlation analysis of expression profiles of *FRG1*, *SRC* and *FMNL2* detected by qRT-PCR and RNA-seq.
