# Supplementary material for: Integrated Analysis Reveals a lncRNA–miRNA–mRNA Network Associated with Pigeon Skeletal Muscle Development
Source: Genes (Basel). 2021 Nov 11;12(11):1787. doi: 10.3390/genes12111787 (PMC8625974; doi:10.3390/genes12111787)
Supplement: Supplementary file 1 [file genes-12-01787-s001.zip › genes-1442823-supplementary material/Table S1.pdf]

1. mireap

perl /export/training/software/mireap_0.2/bin/mireap.pl-i input/smrna.fa -m input/map.txt-r input/ref.fa-o outdir-t test

2. Miranda

-sc 140	Set score threshold to 140
-en -10	Set energy threshold to -10 kcal/mol
-strict	Demand strict 5' seed pairing
-go -4.0	Set gap-open penalty to -4.0
-ge -9.0	Set gap-extend penalty to -9.0

3.TargetScan

Take the 2-8 nt from the 5'end of the small RNA as the 3'-UTR region of seed sequences and transcripts for prediction.
