# Supplementary material for: Integrated Analysis Reveals a lncRNA–miRNA–mRNA Network Associated with Pigeon Skeletal Muscle Development
Source: Genes (Basel). 2021 Nov 11;12(11):1787. doi: 10.3390/genes12111787 (PMC8625974; doi:10.3390/genes12111787)
Supplement: Supplementary file 1 [file genes-12-01787-s001.zip › genes-1442823-supplementary material/Table S2.pdf]

Table S2 The miRNA stem-loop primer and miRNA RT-qPCR primers

miRNA	Reverse transcription primer	QPCR primer
cli-miR-1a-3p	5'--GTCGTATCCAGTGCAGG GTCCGAGGTATTCGCACTG GATACGACATACAT --3'	F: 5'-- GCGCGTGGAATGTAAAGAAGT --3'
		R: 5'-- AGTGCAGGGTCCGAGGTATT --3'
cli-miR-133a-3p	5'--GTCGTATCCAGTGCAGG GTCCGAGGTATTCGCACTG GATACGACACAGCT --3'	F: 5'-- CGTTTGGTCCCCTTCAACC --3'
		R: 5'-- AGTGCAGGGTCCGAGGTATT --3'
cli-miR-133a-5p	5'--GTCGTATCCAGTGCAGG GTCCGAGGTATTCGCACTG GATACGACGATTTG --3'	F: 5'-- CGCGAGCTGGTAAAATGGAAC --3'
		R: 5'-- AGTGCAGGGTCCGAGGTATT --3'

Gene	Primer sequence	Product length
TCONS_00026594	CTGTTTCTGTGGATGCGTGC	144bp
	GAGGACATGACAGCTTGGCT	
TCONS_00066712	GCTTTCCAGGTCCCTTCCAT	105bp
	AGTCAGGACCCAAAACGCAA	
TCONS_00001553	GCCTGTTCCAGTGCTTTGTC	108bp
	CCAATAACAGGGCAAGGGGT	
TCONS_00001557	ACCACCAGCTCTGCTTCTTTT	135bp
	TCAGCTTCACATTCCCATCCC	
TCONS_00003307	CCTTGTCACAGTGGAACCCA	97bp
	CTGATGCGTGTGGACTGGAT	
SRC	GGAGAGCGCCTACAAATCGT	80bp
	TAGCCCGTCTGTCCTGTAGT	
FRG1	GCCGAGCAACTTGACATTGTT	112bp
	TGTCGAGGGCATGGATGTAAG	
FMNL2	GCAGCCCTGGAAAGAGTTGA	85bp
	CCATGGCTTCGTTCTCCGTA	
