# Supplementary material for: Integrated Analysis Reveals a lncRNA–miRNA–mRNA Network Associated with Pigeon Skeletal Muscle Development
Source: Genes (Basel). 2021 Nov 11;12(11):1787. doi: 10.3390/genes12111787 (PMC8625974; doi:10.3390/genes12111787)
Supplement: Supplementary file 1 [file genes-12-01787-s001.zip › genes-1442823-supplementary material/Table S3.pdf]

Group of experiments for Dual luciferase test of TCONS_00026594 and cli-miR-1a-3p

	Group 1	Group 2	Group 3	Group 4	Group 5	Group 6
Vector	pmirGLO	pmirGLO	26594-WT	26594-WT	26594-Mu	26594-Mu
Quantity	1ug	1ug	1ug	1ug	1ug	1ug
Mimics	mimics NC	cli-miR-1a-3p	mimics NC	cli-miR-1a-3p	mimics NC	cli-miR-1a-3p
Quantity	40nM	40nM	40nM	40nM	40nM	40nM

Group of experiments for Dual luciferase test of cli-miR-1a-3p and FRG1

	Group 1	Group 2	Group 3	Group 4	Group 5	Group 6
Vector	pmirGLO	pmirGLO	FRG1-3UTR-WT	FRG1-3UTR-WT	FRG1-3UTR-Mu	FRG1-3UTR-Mu
Quantity	1ug	1ug	1ug	1ug	1ug	1ug
Mimics	mimics NC	cli-miR-1a-3p	mimics NC	cli-miR-1a-3p	mimics NC	cli-miR-1a-3p
Quantity	40nM	40nM	40nM	40nM	40nM	40nM

Group of experiments for Dual luciferase test of cli-miR-1a-3p and SRC

	Group 1	Group 2	Group 3	Group 4	Group 5	Group 6
Vector	pmirGLO	pmirGLO	SRC -3UTR-WT	SRC -3UTR-WT	SRC -3UTR-Mu	SRC -3UTR-Mu
Quantity	1ug	1ug	1ug	1ug	1ug	1ug
Mimics	mimics NC	cli-miR-1a-3p	mimics NC	cli-miR-1a-3p	mimics NC	cli-miR-1a-3p
Quantity	40nM	40nM	40nM	40nM	40nM	40nM

Group of experiments for Dual luciferase test of cli-miR-1a-3p and FMNL2

	Group 1	Group 2	Group 3	Group 4	Group 5	Group 6
Vector	pmirGLO	pmirGLO	FMNL2-3UTR-WT	FMNL2-3UTR-WT	FMNL2-3UTR-Mu	FMNL2-3UTR-Mu
Quantity	1ug	1ug	1ug	1ug	1ug	1ug
Mimics	mimics NC	cli-miR-1a-3p	mimics NC	cli-miR-1a-3p	mimics NC	cli-miR-1a-3p
Quantity	40nM	40nM	40nM	40nM	40nM	40nM
