# Supplementary figures and images for: Integrated Analysis Reveals a lncRNA–miRNA–mRNA Network Associated with Pigeon Skeletal Muscle Development
Source: Genes (Basel). 2021 Nov 11;12(11):1787. doi: 10.3390/genes12111787 (PMC8625974; doi:10.3390/genes12111787)

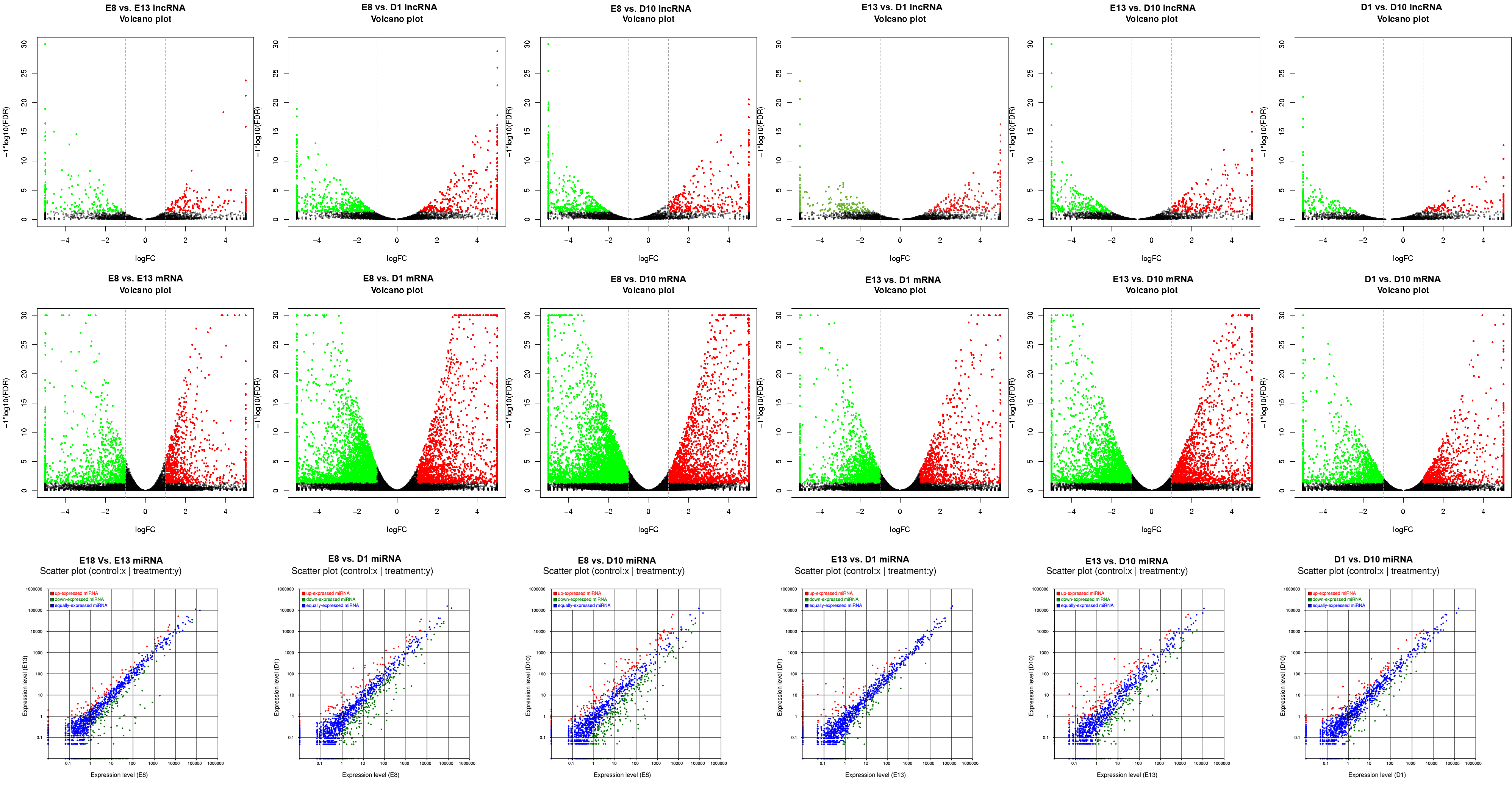

Supplement: Supplementary file 1 [file genes-12-01787-s001.zip › genes-1442823-supplementary material/Figure S1.tif]

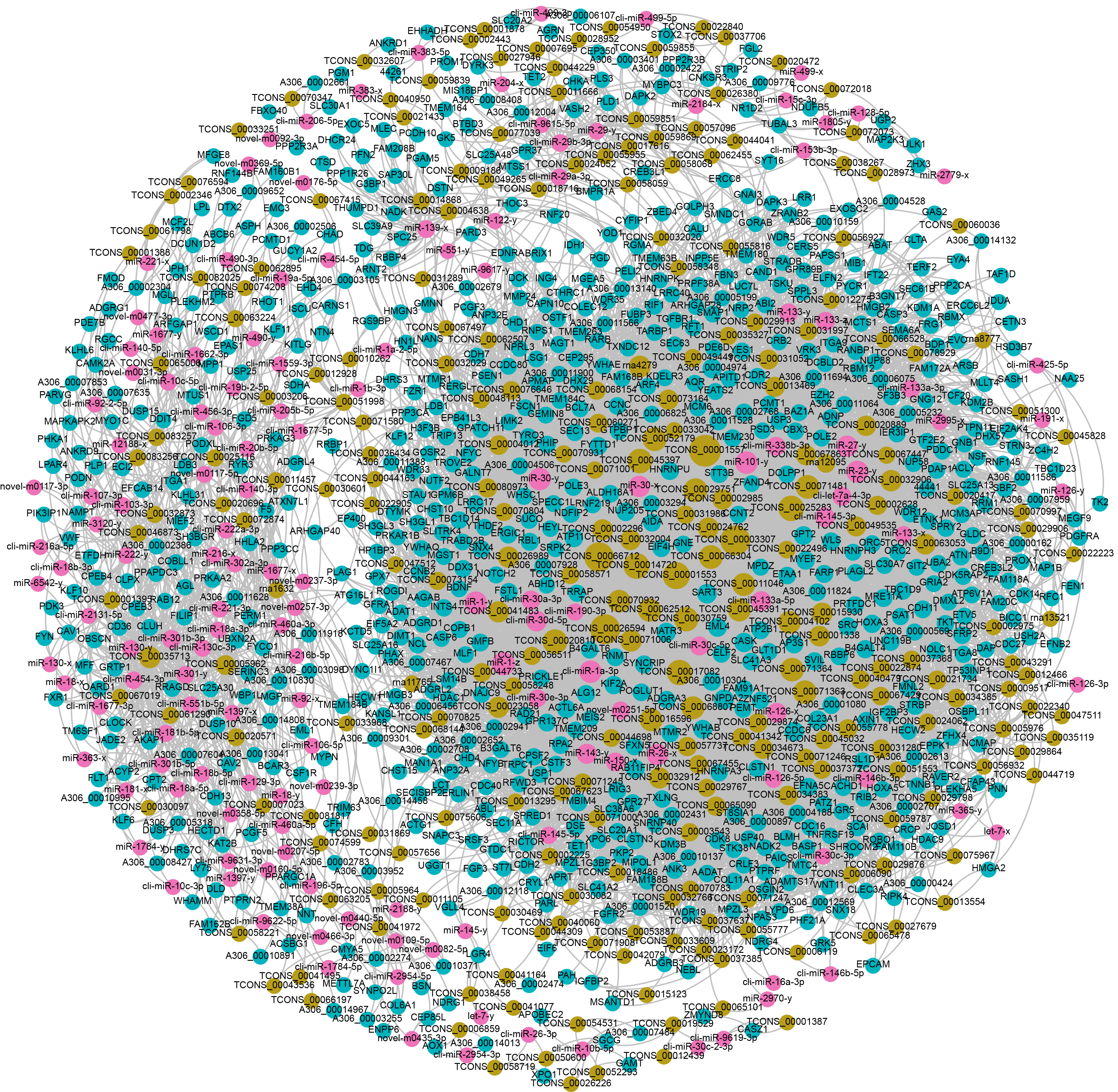

Supplement: Supplementary file 1 [file genes-12-01787-s001.zip › genes-1442823-supplementary material/Figure S2.tif]

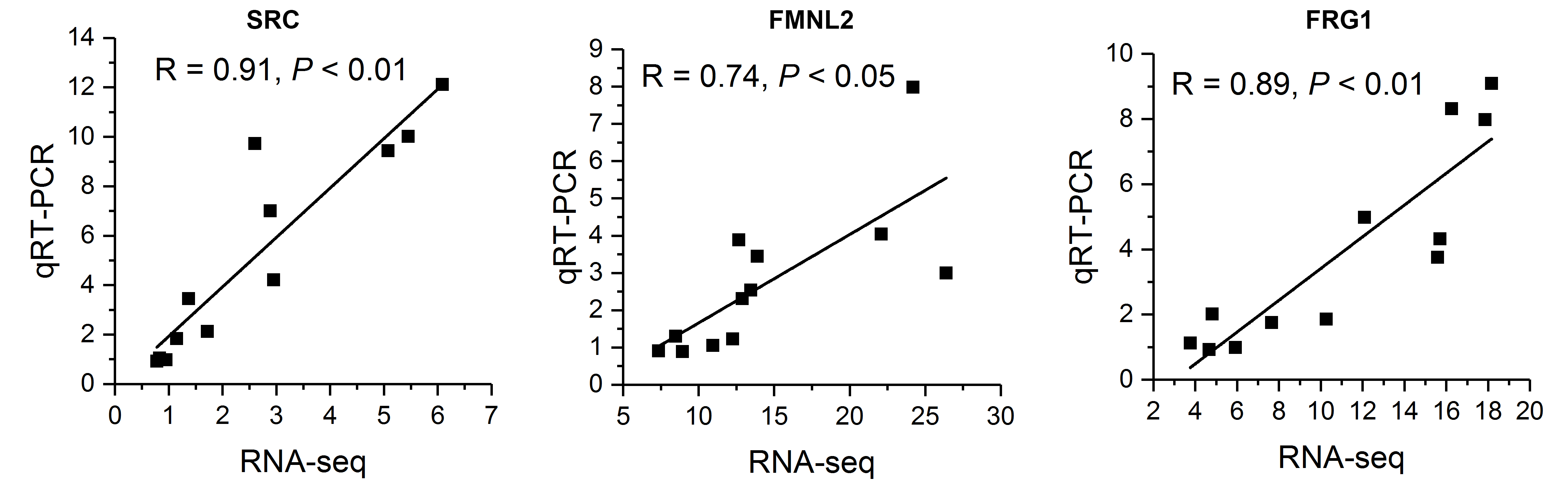

Supplement: Supplementary file 1 [file genes-12-01787-s001.zip › genes-1442823-supplementary material/FigureS3.tif]
